# Characterization of dose impact on IMRT and VMAT from couch attenuation for two Varian couches

**DOI:** 10.1120/jacmp.v12i3.3471

**Published:** 2011-03-02

**Authors:** Heng Li, Andrew K. Lee, Jennifer L. Johnson, Ronald X. Zhu, Rajat J. Kudchadker

**Affiliations:** ^1^ Department of Radiation Physics The University of Texas M. D. Anderson Cancer Center Houston TX; ^2^ Department of Radiation Oncology The University of Texas M. D. Anderson Cancer Center Houston TX USA

**Keywords:** couch attenuation, MV photon radiotherapy, dosimetric impact, QA

## Abstract

In intensity‐modulated radiation therapy (IMRT) and volumetric‐modulated arc therapy (VMAT), the use of posterior oblique beams has become common. Beam attenuation by the treatment couch is not negligible when the couch is in the beam portal. In this study, we established the relationship of relative dose vs. beam angle for two Varian 21EX linacs, one equipped with the Exact couch (standard couch) with sliding side support rails, and the other equipped with the Exact image‐guided radiation therapy (IGRT) carbon fiber couch. Measurements were performed using an ion chamber placed at the center of an acrylic cylindrical phantom positioned at the linac isocenter for 6 MV and 18 MV photon beams. Measurements were performed at three different field sizes (3×3,5×5, and $10\times 10\;{\text{cm}}^{2}$, and were repeated with the phantom positioned at different longitudinal locations on the couches. To evaluate beam attenuation by the standard couch in a clinical setting, two test IMRT plans and two test VMAT plans on the standard couch were delivered. The plans were generated with the sliding rails at the “in” position and delivered with the rails at both “in” and “out” positions. The dose difference to the ion chamber was determined. For oblique fields with 6 MV photons, the standard couch attenuated the radiation beam by up to 26.8%, while the carbon fiber IGRT couch attenuated the beam by up to 4.1%. In the clinical evaluation, the highest dose difference between rails set at the “in” and “out” positions was 2.6% in the IMRT case and 2.1% in the VMAT case. The magnitude of potential dose difference has been quantified and could be used for a quick estimation of dose difference due to couch attenuation in IMRT and VMAT.

PACS number: 87.56.‐v

## I. INTRODUCTION

**Figure 1:**
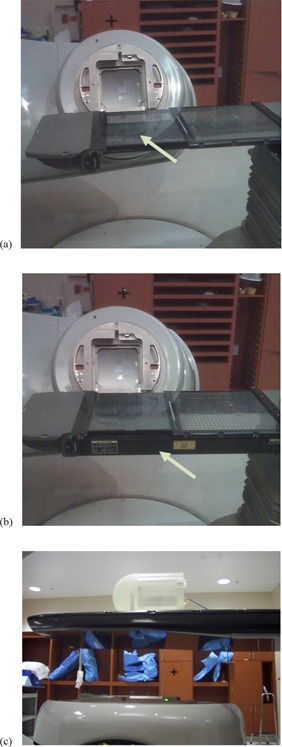
Standard treatment couch with grid insert: (a) sliding rails at center (in) position, (b) sliding rails at side (out) position, with arrows indicating the position of the sliding rail, and (c) IMRT phantom on IGRT carbon fiber couch.

With the growing use of treatment techniques such as intensity‐modulated radiation therapy (IMRT), image‐guided radiation therapy (IGRT), and volumetric‐modulated arc therapy (VMAT), radiation treatment dosimetry is getting more complicated. During IMRT, posterior and/or posterior oblique treatment fields (180° to 270° and 180° to 90° in accordance with the IEC 61217 coordinate system) are routinely used in the treatment of various diseases. In VMAT, one could potentially use arcs with any gantry angle throughout the 360° rotation to achieve a conformal dose distribution. When the beam is incident from posterior oblique angles, the treatment couch can be in the beam portal and, in this instance, it attenuates the beam. In our clinic, two types of treatment couches are used: the standard Varian Exact couch with sliding support couch rails ((Figs. 1a) and (b); Varian Medical Systems, Palo Alto, CA), and the Varian Exact IGRT couch (Fig. 1(c).

For standard couches, the sliding support couch rails can be parked at any position between the center and side (or “in” and “out”) positions (see arrows indicating the position of the sliding rail, (Figs. 1a) and (b)). For more detail structures, refer to Vieira et al.^(1)^. The rails are displaced during IMRT quality assurance (QA) procedure so that they are not in the treatment field. However, during an actual treatment, therapists may or may not move the rails out of the field. The presence of these sliding rails could attenuate the beam and perturb the IMRT fluence for fields passing through the rails. In addition, if imaging for IGRT is being performed on patients just prior to treatment, the sliding rails may be left at the “out” position to avoid obstructing anterior–posterior portal images.

For carbon fiber‐based IGRT couches, several studies^(^ ^2^ ^–^ ^4^ ^)^ have been conducted for the attenuation properties for Siemens couch tops (Siemens Medical Solutions, Concord, CA, USA). The beam attenuation caused by the couch is usually considered small for IMRT;^(^ ^5^ ^–^ ^7^ ^)^ however, Spezi et al.^(8)^ showed in a recent study that an IGRT couch can introduce significant deviation between planning calculations and delivered dose if the couch is not incorporated in the planning process for VMAT. Historically, treatment planning systems have not typically accounted for the presence of the treatment couch. However, some of the latest treatment planning systems incorporate couch modeling for attenuation calculations, and recent studies have shown improved agreement between planning and measured doses when these systems are used.^(^ ^9^ ^,^ ^10^ ^)^

At our institution, two planning systems, Eclipse (Varian Medical Systems, Palo Alto, CA) and Pinnacle (Philips Medical Systems, Milpitas CA) were commissioned and used in the clinic. The Eclipse planning system is used exclusively for RapidArc planning and has the Varian couch models built in, while the Pinnacle system is used for all IMRT planning and couch models are under development.

To fully understand dose differences introduced by treatment couch attenuation in typical IMRT and VMAT treatment delivery and to verify the couch models built into the planning system, we designed and performed a series of tests. These tests included detailed angular measurements for various field sizes and energies and patient plan measurements, to characterize the attenuation of photon beams by standard and IGRT couches. The measurement results could also be used for validation of the couching modeling, as demonstrated in a recently published study by Wagner and Vorwerk.^(11)^

## II. MATERIALS AND METHODS

Measurements were performed on two Varian Clinac 21EX linacs, one of which is equipped with a standard couch and the other with an IGRT couch. The standard couch ((Figs. 1a) and (b)) has two translatable rails and a grid couch insert, all made of carbon fiber, to support the patient.^(1)^ The IGRT couch is composed of carbon fiber, and the thickness of the couch is variable in the superior–inferior direction (Fig. 1(c).

A CIVCO (Kalona, IA) cylindrical acrylic MTQA 1500 phantom (Fig. 1(c) was used in this study. The cylindrical phantom has a diameter of 20 cm and length of 20 cm; it also has a Farmer type ion chamber insert located at the center. Laser alignment was used to position the phantom so that the linac isocenter was coincident with the center of the phantom, and then a Farmer ion chamber (PTW, Freiburg, Germany) was inserted in the phantom to measure the relative dose. The mean of the measured doses at gantry angles of 0°, 90°, and 270° was used as a reference, since there was no beam attenuation by the couch for these beam angles. The gantry was then rotated through angles from 180° to 270° and from 180° to 90°, with measurements taken at 2° increments. These measurements were taken for 6 MV and 18 MV photon beams with field sizes of $3\times 3\;{\text{cm}}^{2},5\times 5\;{\text{cm}}^{2}$, and $10\times 10\;{\text{cm}}^{2}$ on the standard and IGRT couches at two longitudinal positions (at the approximate level of an adult patient's head and pelvis) on each couch. (NB: $3\times 3\;{\text{cm}}^{2}$ was measured for standard couch at pelvis position with 6 MV photon beam only). For the standard couch, measurements were done with the sliding rails parked at the “in” position and with the rails in the “out” position.

The dose difference caused by the standard couch beam attenuation in four typical test treatment plans was also evaluated to determine clinical implications. IMRT treatment plans for one gynecologic (GYN) and one genitourinary (GU) prostate test case and two GU prostate VMAT test cases were used for this analysis. Absolute dose was measured using a CC04 ionization chamber (IBA Dosimetry, Bartlett, TN) inserted into an IMRT QA phantom (IBA Dosimetry, Bartlett, TN) which was placed at the couch location where the pelvis typically would be positioned. This evaluation was accomplished by delivering treatment beams to the IMRT QA phantom with the couch rails set at both the “in” and “out” positions. Each of the two IMRT patient test cases had two oblique beams accounting for ~20% of monitor units. For the two VMAT patient test cases,^(12)^ the couch was modeled with the sliding rails placed at the “in” position in the planning system (Eclipse). For the VMAT test case which was treated with the standard couch, two types of treatment plans were generated. One of the treatment plans utilized two full 360° arcs, while the other utilized two arcs from 225° to 135°, avoiding the posterior angles that intersect the sliding rails. The summary of patient and beam arrangements is listed in Table 1.

**Table 1:** Summary of patient treatment plans and results from the study for the standard couch.

*Patient Treatment Plan*	*Treatment/ Modality*	*Number of Oblique Beams*	*Energy (Oblique Beams)*	*Oblique Beam as % of Total Dose (in MU)*	*Dose Difference (“In” vs. “Out” Rail Positions) (%)*
1	GYN/IMRT	2	18 MeV	17.3	2.6
2	GU/IMRT	2	18 MeV	18.5	2.5
3	GU/VMAT	2×360° arcs	6 MeV	N/A	2.1
4	GU/VMAT	2×225°−135° arcs	6 MeV	N/A	1.1

Abbreviations: MU, monitor unit; GYN, gynecologic; IMRT, intensity‐modulated radiation therapy; GU, genitourinary; VMAT, volumetric modulated arc therapy.

## III. RESULTS

**Figure 2:**
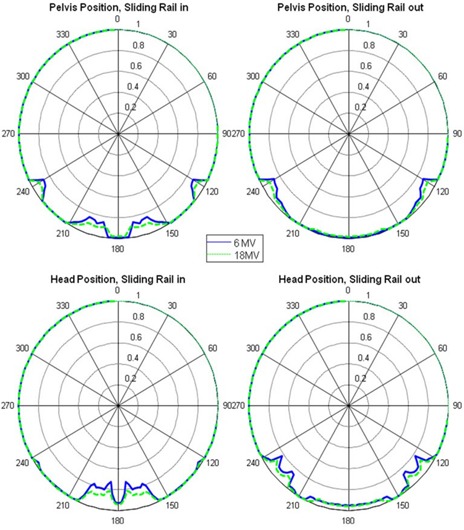
Relative dose versus beam angle for standard couch, field size $5\times 5\;{\text{cm}}^{2}$.

**Figure 3:**
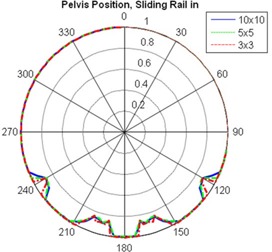
Relative dose versus beam angle for standard couch, 6 MV photon, and field sizes of 3×3,5×5, and $10\times 10\;{\text{cm}}^{2}$.

**Figure 4:**
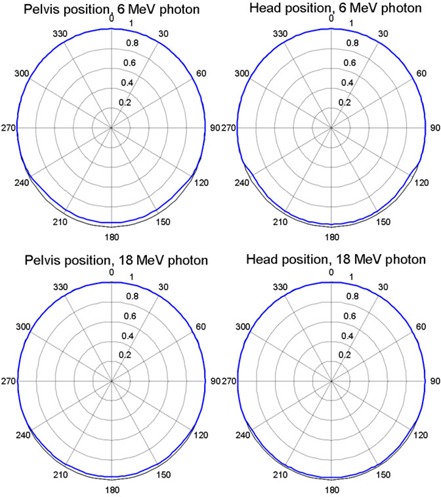
Relative dose versus beam angle for carbon fiber couch, 6 and 18 MV photon, and field size $5\times 5\;{\text{cm}}^{2}$.

Figure 2 shows polar plots of the relative dose (normalized to the mean of the measured doses at gantry angles of 0°, 90°, and 270°) vs. beam angle for the standard couch at field size $5\times 5\;{\text{cm}}^{2}$ and photon beam energies of 6 MV and 18 MV. In general, the 6 MV photon beam yielded more marked dose difference than did the 18 MV photon beam, and the attenuation by the couch was greater for smaller fields than for larger fields. Figure 3 shows polar plots of the relative dose with different field sizes measured with the 6 MV photon, the standard couch, the sliding rail at the “in” position, and the phantom placed at the pelvis location. The minimum relative dose measured was 85.6%, 84.4% and 84.0%, for 10×10,5×5, and $3\times 3\;{\text{cm}}^{2}$ field sizes, respectively. The dose difference introduced by the IGRT carbon fiber couch was more uniform, and for the 6 MV photon beam, it ranged from 3.8% to 4.8% for a $5\times 5\;{\text{cm}}^{2}$ field and from 2.9% to 4.1% for a $10\times 10\;{\text{cm}}^{2}$ field, as shown in Fig. 4.

Table 2 shows the maximum relative dose difference resulting from beam attenuation by both couches at field sizes of $5\times 5\;{\text{cm}}^{2}$, with the standard couch's sliding rails out of the beam portal. The maximum relative dose difference of up to 13.3% for the standard couch (with sliding rails out of the beam portal) was introduced by the frame of the grid couch insert at beam angle between 230° and 250° (as well as between 110° and 130°, Figs. 2 and 3).

**Table 2:** Maximum relative dose error introduced by couch beam attenuation for a field size of $5\times 5\;{\text{cm}}^{2}$ (with sliding rails out of the beam portal for the standard couch).

*Energy*	*Couch Type*	*Position*	*Maximum Dose Error (%)*
	Standard	Head	5.0
		Pelvis	13.3
6 MV			
	IGRT	Head	3.9
		Pelvis	4.8
	Standard	Head	3.6
		Pelvis	8.3
18 MV			
	IGRT	Head	2.2
		Pelvis	3.2

Abbreviation: IGRT, image‐guided radiation therapy.

The maximum relative dose differences for the standard couch with the sliding rails in the beam portal are listed in Table 3. The sliding rails introduced a dose difference of up to 26.8% for the posterior–anterior oblique beam at the head position (Table 3).

**Table 3:** Maximum relative dose error introduced by couch beam attenuation for the standard couch for field sizes of $5\times 5\;{\text{cm}}^{2}$ and $10\times 10\;{\text{cm}}^{2}$ (with sliding rails in the beam portal).

*Energy*	*Field Size*	*Position*	*Maximum Dose Error (%)*
	$5\times 5\;{\text{cm}}^{2}$	Head	26.8
		Pelvis	15.6
6 MV			
	$10\times 10\;{\text{cm}}^{2}$	Head	25.2
		Pelvis	14.4
	$5\times 5\;{\text{cm}}^{2}$	Head	17.6
		Pelvis	9.7
18 MV			
	$10\times 10\;{\text{cm}}^{2}$	Head	16.5
		Pelvis	9.2

For the two IMRT and two VMAT test treatment plans delivered to the phantom, the maximum dose difference was 2.6% between treatments delivered with the sliding rails in the “in” position and those delivered with the rails in the “out” position, as shown in Table 1. The dose differences were referenced to the “in” position.

## IV. DISCUSSION

Our results showed that the standard couch beam attenuation and the dose difference introduced by the attenuation, were strongly angular‐dependent, which, in turn, was affected by the position of the sliding rails. The sliding rails heavily attenuated the photon beam. With the sliding rails in the beam portal (Table 3), the maximum dose errors for 6 MV photon beam and a 10×10 field are 25.2% and 14.4% for head and pelvis location, respectively. These results are comparable with Wagner and Vorwerk^(11)^ (maximum 17.1% for $10\times 10\;{\text{cm}}^{2}$ field with 5° increment measurements). However, the dose difference of the beam at other beam angles that were not attenuated by the rails was around 1%. For the IGRT couch, the angular dependence of beam attenuation and dose difference was not as strong; for 6 MV photon beams at the pelvis location (Table 2 and Fig. 4), it ranged from 3.8% to 4.8% for a $5\times 5\;{\text{cm}}^{2}$ field and from 2.9% to 4.1% for a $10\times 10\;{\text{cm}}^{2}$ field, which was consistent with Vanetti et al.^(9)^ (from 3.1% to 4.4% for a $10\times 10\;{\text{cm}}^{2}$ field). The angular dependence of beam attenuation by the IGRT couch could be due to the difference in the lengths of beam paths through the couch at different beam angles.

One possible perceived limitation of this study was that the measurements were performed on two different linacs. However, the two linacs had matching beam data (including energy spectrum) when they were commissioned and thus were considered to be identical for the purposes of this study. Although there could be minor deviations in percentage dose depth, output, or beam energy between the two linacs, our results and conclusion are not affected by these minor deviations. Output of the linacs was measured in annual QA tests at 0°, 90°, 180°, and 270°, and the variation in dose due to measurement angle was determined to be < 1% for both linacs.

The dose difference introduced by attenuation through both linac couches also showed dependency on field size, entry position of the beam, and photon beam energy, as illustrated in Fig. 3 and Tables 2 and 3. Attenuation of the photon beam was slightly higher for 3×3 and $5\times 5\;{\text{cm}}^{2}$ fields than for $10\times 10\;{\text{cm}}^{2}$ fields (The minimum relative dose measured was 84.0%, 84.4% and 85.6%, respectively, for sliding rail at the “in” position, 6 MV and pelvis location). However, the smaller field size is more sensitive to angular change of the gantry; a small gantry angle change could lead to larger dose difference for $3\times 3\;{\text{cm}}^{2}$, as shown in Fig. 3. This is due to the fact that the beam opening for a $3\times 3\;{\text{cm}}^{2}$ field (1.7°) is smaller than that for the $10\times 10\;{\text{cm}}^{2}$ field (5.7°). The attenuation of 6 MV photon beams was higher than that of 18 MV beams (maximum dose difference 14.4% vs. 9.2% at the head position for $10\times 10\;{\text{cm}}^{2}$ fields). The dose differences at different measurement points on the couch (i.e., head and pelvis) was negligible for the IGRT couch; however, because of the design of the standard couch (Fig. 1), the positioning (head vs. pelvis) could also affect the magnitude and angular distribution of beam attenuation by the couch.

For the two IMRT patient treatment plans which had two oblique beams accounting for ~ 20% of monitor units, we observed an ~ 2.5% dose difference to the ion chamber between treatments delivered with the sliding rails at the “in” position and those delivered with the rails at the “out” position. For the VMAT test treatment plans delivered with rails set at “in” and “out” positions, the dose differences between treatment plans delivered with rails set at both these positions were 2.1% and 1.1% for the 360° and 270° (from 225° to 135°) arcs, respectively, as shown in Table 1. Based on these patient plan measurements, ~ 2% dose difference could be expected if the treatment planning systems were not commissioned to take into account the couch modeling correctly, or the treatment was not carried out as planned. There are several contributing factors to the dose difference: 1) dose difference due to rails in the beam portal being sensitive to the beam angle, 2) 18 MeV were used for posterior oblique gantry angles in our IMRT test cases, and 3) only ~ 20% MU are from the posterior oblique beams for our test cases. Therefore, although in the worse case, the dose difference could be estimated to be up to 5% (worse beam angle, 6 MV, 20% MU), we measured a maximum dose difference of 2.6% for our clinical cases. If the couch model is not implemented in the planning system, we recommend avoiding beam angles between 230° and 250° (as well as between 110° and 130°, Figs. 2 and 3) for the Varian standard couch.

In addition to lowering the treatment dose from certain beam angles to the target area of the patient, the treatment couch increases the skin dose because of the buildup effect, as shown in literature.^(^ ^13^ ^–^ ^15^ ^)^

## V. CONCLUSIONS

Dose difference resulting from beam attenuation by the treatment couch could be clinically significant for patients treated with posterior and/or oblique posterior photon beams, particularly those patients treated with smaller fields, such as those used in IMRT. We have characterized the dose difference due to couch attenuation of two Varian couches through detailed angular measurements with different field sizes and energies, and demonstrated that for IMRT or VMAT, an ~ 2% dose difference could be expected for GYN or GU cases if the treatment couch was not included in the planning system or the treatment was not carried out consistently with the plan. Our results further indicate that IMRT QA tests should be performed with the couch rails in exactly the same position used during patient treatment. This practice would help ensure that the QA results reflect the dose the patient actually receives. If possible, couch parameters or corrections should be included in treatment planning systems, and beam angles should be carefully selected to avoid underdosing patients.

## ACKNOWLEDGMENTS

We wish to thank Bryan Tutt of the Department of Scientific Publications of M. D. Anderson Cancer Center for his editorial review of this manuscript.

## References

[1] Vieira SC , Kaatee RS , Dirkx ML , Heijmen BJ . Two‐dimensional measurement of photon beam attenuation by the treatment couch and immobilization devices using an electronic portal imaging device. Med Phys. 2003;30(11):2981–87. 14655945 10.1118/1.1620491

[2] Meydanci TP and Kemikler G . Effect of a carbon fiber tabletop on the surface dose and attenuation for high‐energy photon beams. Radiat Med. 2008;26(9):539–44. 19030962 10.1007/s11604-008-0271-6

[3] Spezi E , Ferri A . Dosimetric characteristics of the Siemens IGRT carbon fiber tabletop. Med Dosim. 2007;32(4):295–98. 17980831 10.1016/j.meddos.2006.11.006

[4] Poppe B , Chofor N , Rühmann A , et al. The effect of a carbon‐fiber couch on the depth‐dose curves and transmission properties for megavoltage photon beams. Strahlenther Onkol. 2007;183(1):43–48. 17225945 10.1007/s00066-007-1582-8

[5] Myint WK , Niedbala M , Wilkins D , Gerig LH . Investigating treatment dose error due to beam attenuation by a carbon fiber tabletop. J Appl Clin Med Phys. 2006;7(3):21–27. 10.1120/jacmp.v7i3.2247 PMC5722426 17533341

[6] McCormack S , Diffey J , Morgan A . The effect of gantry angle on megavoltage photon beam attenuation by a carbon fiber couch insert. Med Phys. 2005;32(2):483–87. 15789595 10.1118/1.1852792

[7] Njeh CF , Raines TW , Saunders MW . Determination of the photon beam attenuation by the Brainlab imaging couch: angular and field size dependence. J Appl Clin Med Phys. 2009;10(3):2979. 10.1120/jacmp.v10i3.2979 PMC5720553 19692980

[8] Spezi E , Angelini AL , Romani F , et al. Evaluating the influence of the Siemens IGRT carbon fiber tabletop in head and neck IMRT. Radiother Oncol. 2008;89(1):114–22. 18692264 10.1016/j.radonc.2008.06.011

[9] Vanetti E , Nicolini G , Clivio A , Fogliata A , Cozzi L . The impact of treatment couch modelling on RapidArc. Phys Med Biol. 2009;54(9):N157–N166. 19351984 10.1088/0031-9155/54/9/N03

[10] Mihaylov IB , Corry P , Yan Y , Ratanatharathorn V , Moros EG . Modeling of carbon fiber couch attenuation properties with a commercial treatment planning system. Med Phys. 2008;35(11):4982–88. 19070232 10.1118/1.2982135

[11] Wagner D and Vorwerk H . Treatment couch modeling in the treatment planning system Eclipse. J Cancer Sci Ther. 2011;3:188–93.

[12] Otto K . Volumetric modulated arc therapy: IMRT in a single gantry arc. Med Phys. 2008;35(1):310–17. 18293586 10.1118/1.2818738

[13] Lee KW , Wu JK , Jeng SC , Hsueh Liu YW , Cheng JC . Skin dose impact from vacuum immobilization device and carbon fiber couch in intensity modulated radiation therapy for prostate cancer. Med Dosim. 2009;34(3):228–32. 19647634 10.1016/j.meddos.2008.10.001

[14] De Ost B , Vanregemorter J , Schaeken B , Van den Weyngaert D . The effect of carbon fibre inserts on the build‐up and attenuation of high energy photon beams. Radiother Oncol. 1997;45(3):275–77. 9426122 10.1016/s0167-8140(97)00118-7

[15] Higgins DM , Whitehurst P , Morgan AM . The effect of carbon fiber couch inserts on surface dose with beam size variation. Med Dosim. 2001;26(3):251–54. 11704460 10.1016/s0958-3947(01)00071-1

